# Meta-analysis of neoadjuvant chemotherapy compared to radical cystectomy alone in improving overall survival of muscle-invasive bladder cancer patients

**DOI:** 10.1186/s12894-020-00733-z

**Published:** 2020-10-14

**Authors:** Agus Rizal A. H. Hamid, Fanny Riana Ridwan, Dyandra Parikesit, Fina Widia, Chaidir Arif Mochtar, Rainy Umbas

**Affiliations:** 1 Urology Department, Faculty of Medicine, Universitas Indonesia, Cipto Mangunkusumo Hospital, Jakarta, Indonesia; 2 Urology Department, Faculty of Medicine, Universitas Indonesia, Universitas Indonesia Hospital, Depok, West Java Indonesia

**Keywords:** Neoadjuvant chemotherapy, Radical cystectomy, Muscle-invasive bladder cancer, Overall survival

## Abstract

**Background:**

Most patients with muscle-invasive bladder cancer (MIBC) developed metastasis within 2 years, even after radical cystectomy (RC). The recurrence rate of MIBC was more than 50% of the cases. A meta-analysis conducted by Yin et al. showed that neoadjuvant chemotherapy (NAC) + RC improves overall survival in MIBC compared with RC only. However, a new meta-analysis by Li et al. concluded that NAC + RC was not superior to RC only in improving overall survival. The inconsistencies of these studies required further comprehensive analysis to recommend NAC use in bladder cancer treatment. Therefore, this meta-analysis aims to analyze previous studies that compare the efficacy of NAC + RC versus RC only to improve overall survival of MIBC.

**Methods:**

The articles were searched using Pubmed with keywords “muscle-invasive bladder cancer”, “neoadjuvant chemotherapy”, “cystectomy”, and “overall survival”. The articles that were published until June 2020 were screened. The overall survival outcome was analyzed as hazard ratio (HR) and presented in a forest plot.

**Result:**

Seventeen studies were included in meta-analysis with a total sample of 13,391 patients, consist of 2890 received NAC followed by RC and 10,418 underwent RC only. Two studies used methotrexate/vinblastine/doxorubicin/cisplatin (MVAC), two studies used gemcitabine/cisplatin (GC), one study used Cisplatin-based regimen, one study used MVAC or GC, one study used gemcitabine/carboplatin (GCarbo) or GC or MVAC, one study used Cisplatin/Gemcitabine or MVAC, one study used Cisplatin only, one study used Cisplatin-based (GC, MVAC) or non-Cisplatin-based (combined paclitaxel/gemcitabine/carboplatin), one study used GC, MVAC, Carboplatin, or Gemcitabine/Nedaplatin (GN), and five studies did not mention the regimen The overall survival in the NAC + RC only group was significantly better than the RC only group (HR 0.82 [0.71–0.95], *p* = 0.009).

**Conclusion:**

NAC + RC is recommended to improve overall survival in MIBC patients. A further study assessing side effects and quality of life regarding NAC + RC is needed to establish a strong recommendation regarding this therapy.

## Background

Bladder cancer is the ninth most common malignancy worldwide. The incidence of bladder cancer in men are three times more frequently than women [1]. The regions with the highest incidence of bladder cancer were Southern and Western Europe, North America, Northern Africa, and Western Asia [1]. The 5-year survival rates of bladder cancer patients vary from 97% (stage I) to 22% (stage IV) [2]. Occult metastasis at the time of diagnosis is the main reason why muscle-invasive bladder cancer (MIBC) has a poor prognosis [3]. Even after radical cystectomy (RC), MIBC mostly develops within 2 years with a recurrence rate of more than 50% of cases [2].

Studies have been conducted to find supportive treatments that improved survival rate in patients with MIBC, such as perioperative chemotherapy and radiotherapy. Since radiotherapy has unclear efficacy to improve survival in MIBC [4], the role of chemotherapy is commonly studied in recent years. Transitional cell carcinoma of the bladder (TCCB), the most common pathological type of bladder cancer [5], is a chemosensitive disease that responds to cisplatin-based regimens, with responses varies from 50 to 70% in the metastatic state [6]. This makes chemotherapy a promising additional treatment in MIBC. Chemotherapy in MIBC can be administered preoperatively (neoadjuvant) and postoperatively (adjuvant) [6]. The main reason for neoadjuvant chemotherapy (NAC) is to treat micrometastatic disease at the time of diagnosis when the disease burden is at its lowest [7]. Petrelli et al. stated that the use of NAC caused MIBC downstaging [8]. The lesion response after NAC may be used as a predictive factor of long-term survival [8]. Patients may tolerate chemotherapy better before surgery compared to postoperative chemotherapy [6]. Seah et al. showed that despite the development of various surgical techniques, 64% of patients had complications within 90 days after RC, resulting the inability to administer postoperative chemotherapy in up to 30% of these patients [9].

Previous studies proved that NAC + RC improves overall survival in MIBC compared with radical cystectomy alone. In 2012, a meta-analysis that included 11 RCTs showed 5% absolute improvement in overall survival at 5 years (HR 0.86 [0.77–0.95]) [10]. However, another meta-analysis conducted in 2017 showed that NAC + RC was consistent with no difference to cystectomy or radiotherapy alone [2]. The inconsistencies of these studies require further comprehensive analysis to recommend NAC use in MIBC treatment. Therefore, this meta-analysis aims to analyze previous studies that compare the efficacy of NAC + RC versus RC only to improve overall survival of MIBC.

## Methods

This meta-analysis was conducted according to PRISMA guidelines. The articles were searched using Pubmed with keywords “muscle-invasive bladder cancer”, “neoadjuvant chemotherapy”, “cystectomy”, and “overall survival”. Articles that were published until June 2020 were screened. The inclusion criteria were (1) MIBC patients proven by histology examination; (2) Two-arm studies that compare NAC + RC and RC only, (3) No distant metastases; and (4) Studies with overall survival outcome. The exclusion criteria were (1) Studies that compared different NAC regimens without RC; (2) Single-arm studies; (3) Case reports, reviews, commentaries; (4) Animal studies; and (5) Non-English written studies.

The information of author, year of publication, total samples, NAC regimens, clinical stage, and mean length of follow up period were collected from the included studies. The meta-analysis was conducted using Revman 5.3 (Cochrane, Oxford, U.K.). The overall survival outcome was analyzed as hazard ratio (HR) and presented in a forest plot. The heterogeneity was assessed using χ^2^-based test, I^2^ and Q statistics. The initial analysis is performed with fixed-effect model. If there are significant heterogeneities, the analysis will be carried out using random-effect model. The pooled effect was determined with *p* < 0.05 was considered statistically significant. Sensitivity test was performed by excluding one study from the meta-analysis.

## Results

**Fig. 1:**
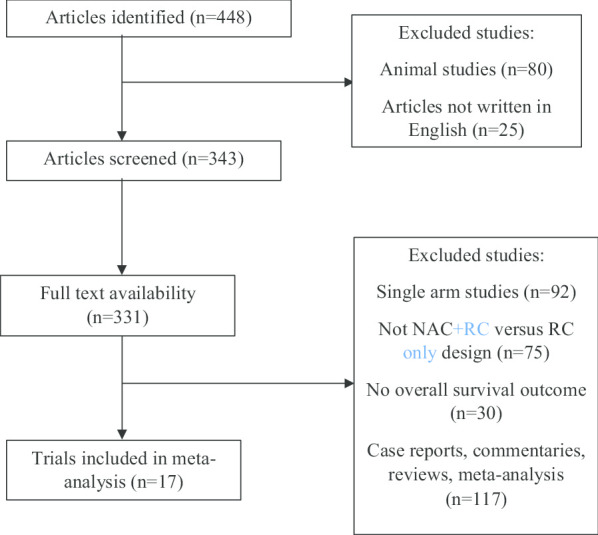
Literature searching flow chart

The flowchart of literature searching is shown in Fig. 1. A total of 448 articles were identified using the keywords. Animal studies (n = 80) and articles not written in English (n = 25) were excluded from literature searching. Articles that were available in full text were included (n = 331). Seventeen studies were included in this meta-analysis.

There was a total of 13,391 patients reviewed in this meta-analysis with varied sample size, from 60 to 2018 patients. The age range of the subjects was 48.9 to 84 years old. A total of 2890 patients received NAC followed by RC and 10,418 patients underwent RC alone. The clinical stages of patients included were T2–T4. Two studies used methotrexate/vinblastine/doxorubicin/cisplatin (MVAC), two studies used gemcitabine/cisplatin (GC), one study used Cisplatin-based regimen, one study used MVAC or GC, two study used gemcitabine/carboplatin (GCarbo) or GC or MVAC, one study used Cisplatin/Gemcitabine or MVAC, and one study used Cisplatin only. One study used Cisplatin-based (GC, MVAC) or non-Cisplatin-based (combined paclitaxel/gemcitabine/carboplatin (PGC) or GCarbro) and one study used GC, MVAC, Carboplatin or Gemcitabine/Nedaplatine (GN). Five studies did not mention the chemotherapy regimen, and one study mentioned that the data was not available on the registry. The characteristics of studies are described in Table 1.

**Table 1:** Characteristics of included studies

References	Type	Number of Samples		NAC regimen	Median survival (months)	
		NAC + RC	RC Only		NAC + RC	RC Only
MVAC						
Kitamura et al. [11]	RCT	64	66	MVAC	102	82
Grossman et al. [12]	RCT	153	154	MVAC	77	46
GC						
Khaled et al. [13]	RCT	59	59	GC	NA	NA
Osman et al. [14]	Clinical trial	30	30	GC	36	28
Other regimens						
Lane et al. [15]	Retrospective cohort	381	1505	Cisplatin-based	NA	NA
Anan et al. [16]	Retrospective cohort	336	196	GCarbo, GC, MVAC	NA	NA
Hinata et al. [17]	Retrospective cohort	69	69	GC or MVAC	NA	NA
Martinez-Pineiro et al. [18]	RCT	62	60	Cisplatin monotherapy	NA	NA
Milenkovic et al. [19]	Retrospective cohort	102	389	NA	106.7	60.1
Mozzane et al. [20]	Retrospective cohort	1519	2459	NA	NA	NA
Gronostaj et al. [21]	Retrospective cohort	79	76	Cisplatin-based, GCarbo	NA	NA
Boeri et al. [22] (2)	Retrospective cohort	156	166	Cisplatin-based (GC, MVAC), noncisplatin-based (combined PGC, or GCarbo)	NA	NA
Nitta et al. [23]	Retrospective cohort	69	71	GC, MVAC, carboplatin, GN	NA	NA
Ploussard et al. [24]	Retrospective cohort	56	394	MVAC, GC	16.7	28.8
Russell et al. [25]	Retrospective cohort	216	216	No data available in BladderBase	NA	NA
Vetterlein et al. [26]	Retrospective cohort	369	1649	NA	51.7	29
Hermans et al. [27] (1)	Retrospective cohort	191	4164	NA (cT2 disease)	NA	NA
Hermans et al. [27] (2)	Retrospective cohort	133	834	NA (cT3-4 disease)	NA	NA

We also conducted a risk of bias using Cochrane Risk of Bias Assessment Tools for RCT (Table 2) and Newcastle–Ottawa Assessment Tools for cohort studies (Table 3).

**Table 2:**
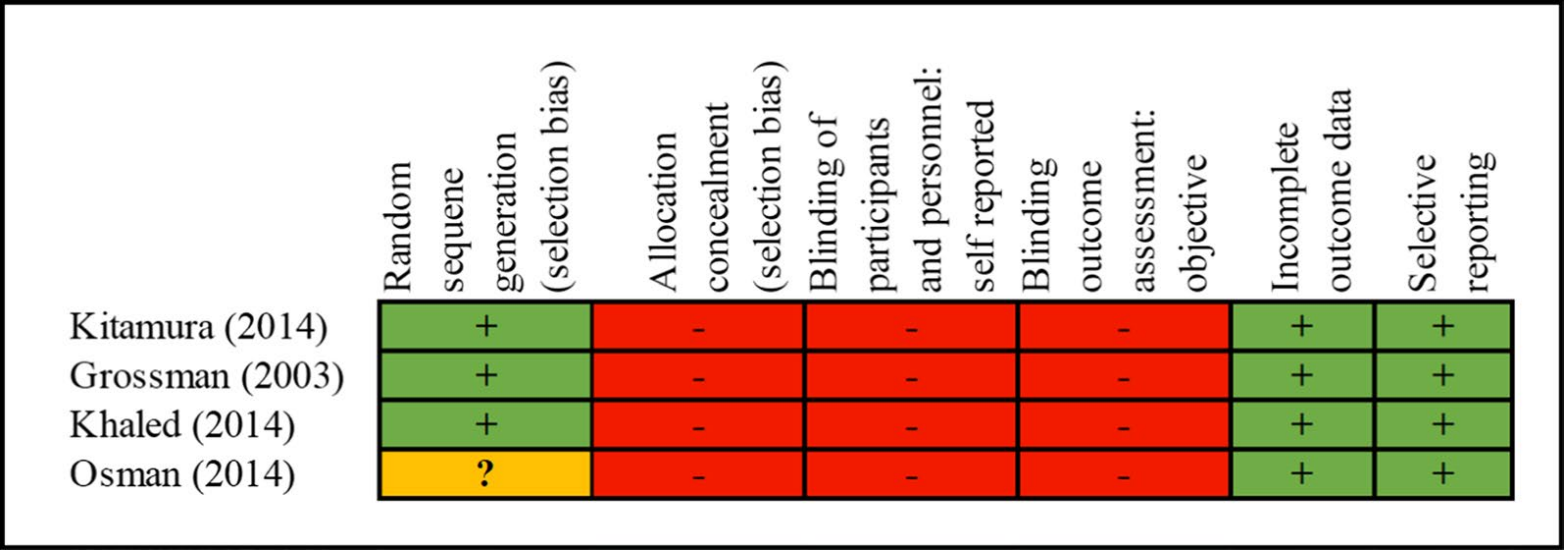
Risk of bias for clinical trials using Cochrane Risk of Bias Tools

**Table 3:** Risk of bias for cohort studies using Newcastle–Ottawa Assessment tools

Study	Selection				Comparability	Outcome			Overall
	Representativeness of exposed cohort	Selection of nonexposed	Ascertainment of exposure	Outcome not present at start	Study controls for treatment	Assessment of outcome	Adequate follow-up length	Adequacy of follow-up	
Lane (2018)	*a	*	*	*	*d	*	*	*	8
Anan (2017)	*b	*	*	*	-	*	*	*	7
Hinata (2017)	*b	*	*	*	*e	*	*	*	8
Martinez-Pineiro (1995)	*b	*	*	*	*f	*	*	*	8
Milenkovic (2018)	-c	*	*	*	-g	*	*	*	6
Mozzane (2019)	*a	*	*	*	*d,e	*	*	*	8
Gronostaj (2019)	-c	*	*	*	-g	*	*	*	6
Boeri (2019)	-a	*	*	*	*e	*	*	*	7
Nitta (2019)	*b	*	*	*	-g	*	*i	*	7
Ploussard (2020)	*b	*	*	*	–	*	*	*	7
Russell (2019)	*a	*	*	*	*e	*	*	*	8
Vetterlein (2017)	*a	*	*	*	*d	*	*	*	8
Hermans (2019)	*a	*	*	*	-h	*	*	*	7

^a^National or international registry

^b^More than 1 institution

^c^Single institution

^d^Adjust age, sex, race, and other factors

^e^Adjust with propensity score matched analyses

^f^No significant differences for age, size, and performance status

^g^Not controlled, age showed significant difference

^h^Not possible to do propensity score match analysis for potential confounders and important prognostic factor

^i^Significant shorter follow-up period in treatment group

Due to the cancer treatment which consisted of neoadjuvant chemotherapy and surgery, both the clinicians and patients were informed about the treatment and all the patients provided with written informed consent. Thus, a high risk of bias of allocation concealment and blinding process could not be avoided.

Six studies had no star in comparability domain; thus, it had poor quality. Other studies considered as good quality as they met the requisite of minimum of 3 stars in selection domain, one star in comparability domain, and three stars in outcome domain.

**Fig. 2:**
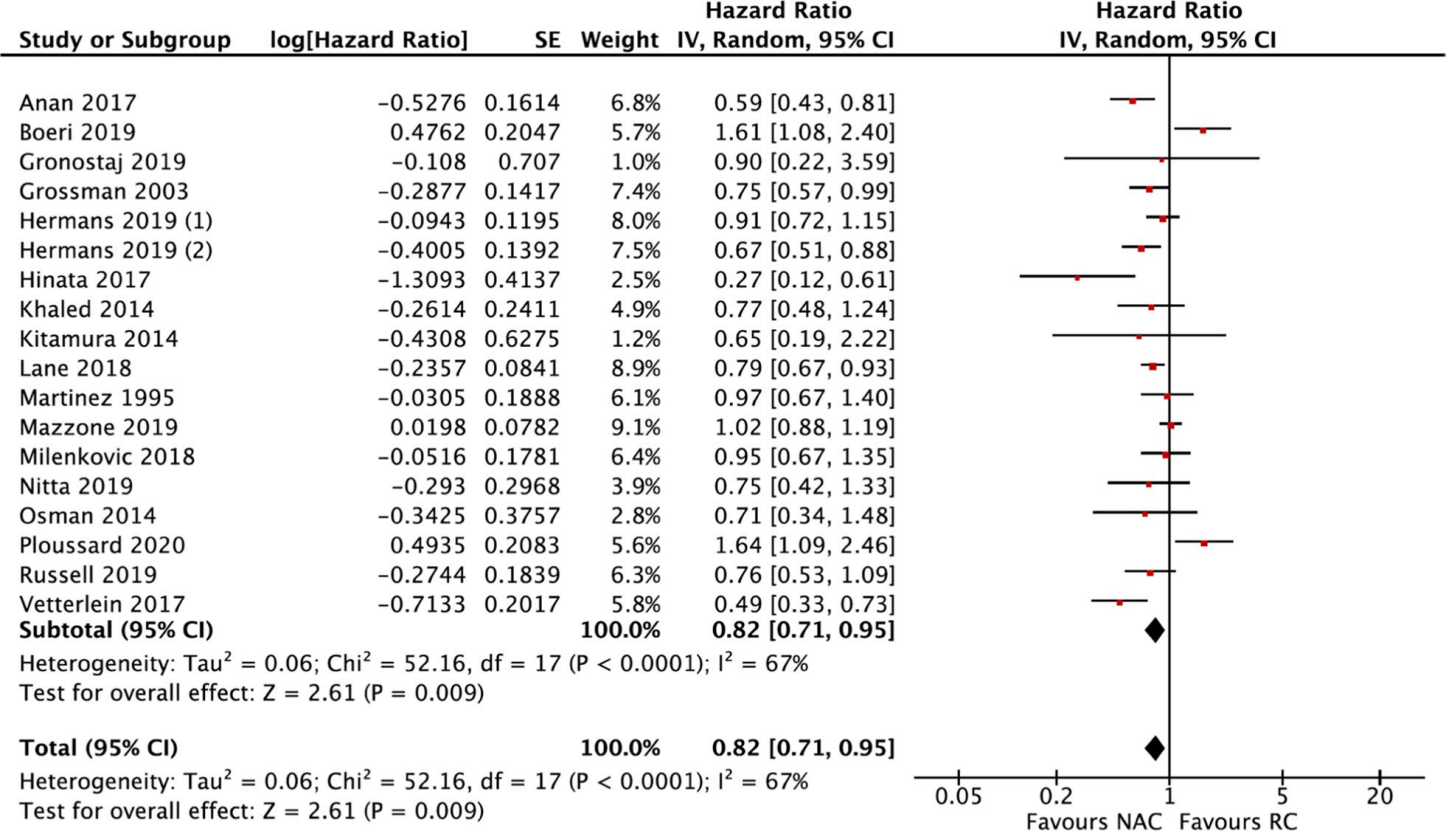
Forest plot of included studies

All studies were included in the forest plot with initial analysis performed using random-effect model. The random effect model was used due to significantly substantial heterogeneity in fixed models (I^2^ = 67%, *p* < 0.0001). The random effect model resulted in the same manner with I^2^ = 67% and *p* < 0.0001. The overall survival in the NAC + RC group was significantly better than the RC only group (HR 0.82 [0.71–0.95], *p* = 0.009) as showed in Fig. 2.

**Fig. 3:**
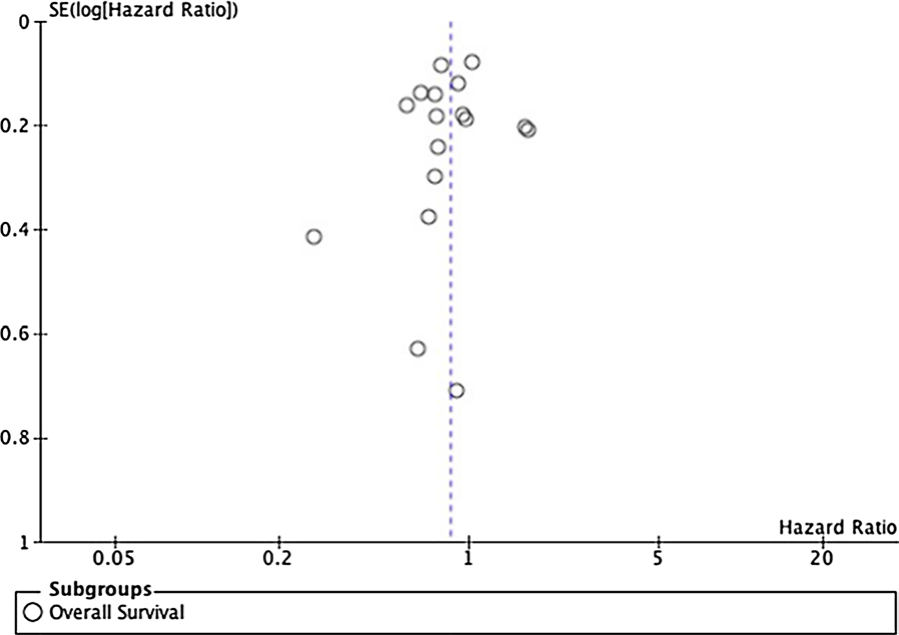
Funnel plot of overall survival for publication bias

We included more than ten studies, so we decided to analyze the treatment effects estimated from individual studies against a measure of study size, as well as to assess and detect a publication bias, using funnel plot. The funnel plot was shown in Fig. 3. The plot is considered asymmetrical with missing studies on the middle and bottom right of the plot. Study by Gronostaj et al. filled in the bottom part of the plot due to its smallest study and largest standard error (0.707). In contrast, study by Mazzone et al., which lies on the top of the plot, had a maximum overall score with proper adjustment of other factors that might influenced the result, which resulted in smallest standard error. X-axis represented the mean result of the hazard ratios, which lays left to the one, favoring the NAC + RC group. We used a random effect model; thus, the triangle cannot be shown.

Sensitivity analysis with one study removed was performed and significant P value in all studies was shown in Table 4.

**Table 4:** Sensitivity analysis of overall survival

References	Statistics with study removed				
	HR	Lower limit	Upper limit	Z-value	*P* value
Lane et al. [15]	0.82	0.70	0.97	2.31	0.02
Anan et al. [16]	0.84	0.73	0.98	2.24	0.02
Hinata et al. [17]	0.85	0.74	0.97	2.35	0.02
Osman et al. [14]	0.83	0.71	0.96	2.50	0.01
Khaled et al. [13]	0.82	0.71	0.96	2.47	0.01
Kitamura et al. [11]	0.82	0.71	0.96	2.54	0.01
Grossman et al. [12]	0.83	0.71	0.97	2.37	0.02
Martinez et al. [18]	0.81	0.70	0.95	2.639	0.009
Milenkovic et al. [19]	0.81	0.70	0.95	2.61	0.009
Mazzone et al. [20]	0.80	0.69	0.94	2.72	0.007
Boeri et al. [22]	0.79	0.69	0.91	3.27	0.001
Gronostaj et al. [21]	0.82	0.71	0.95	2.59	0.01
Hermans et al. [27] (1)	0.81	0.69	0.95	2.53	0.01
Hermans et al. [27] (2)	0.84	0.72	0.97	2.29	0.02
Nitta et al. [23]	0.83	0.71	0.96	2.48	0.01
Ploussard et al. [24]	0.79	0.69	0.91	3.28	0.001
Russell et al. [25]	0.83	0.71	0.96	2.42	0.02
Vetterlein et al. [26]	0.85	0.74	0.98	2.22	0.03

There were 3 studies with adverse effects data of the NAC + RC group. The most prevalent adverse effect was granulocytopenia (moderate 18.60%, severe 23.25%), followed by nausea or vomiting (moderate 25.55%, severe 10.21%, life-threatening 0.47%) and stomatitis (moderate 8.02%). The adverse effects were listed in Table 5.

**Table 5:** Adverse effects in NAC group

Adverse effects	Khaled et al. [13] (n = 59)	Grossman et al. [12] (n = 153)	Pineiro et al. [18] (n = 62)
	Moderate/severe/life-threatening		
Granulocytopenia	NA	35/50/0	5/0/0
Nausea or vomiting	20/2/NA	9/0/0	41/26/1
Stomatitis	2/0/NA	15/0/0	NA
Anemia	2/0/NA	9/1/0	3/0/0
Thrombocytopenia	3/0/NA	7/0/0	5/0/0
Diarrhea or constipation	1/0/NA	6/0/0	NA
Neutropenia	2/0/0	2/0/NA	NA
Fatigue, lethargy, and malaise	NA	5/0/0	NA
Neuropathy	NA	3/0/0	3/0/0
Renal effects	NA	1/0/0	3/0/0

## Discussion

Most of the studies included in this meta-analysis showed improved overall survival in the NAC group except for three studies. First, a study by Ploussard et al. found that NAC patients had a 1.6-fold higher risk to death (HR 1.638[1.089–2.465], *p* = 0.018) [24]. Second, a study by Boeri et al. when using both suboptimal dose (HR 1.71[1.08–2.69], *p* = 0.02) and no NAC (HR 1.61[1.06–3.01], *p* = 0.02), compared to optimal NAC group [22]. Then, a study by Mazzone et al. which showed that NAC was no longer associated with lower overall mortality (HR 1.02[0.90–1.16], *p* = 0.8) [20].

Based on the current meta-analysis using random effect model, the overall survival in the NAC + RC group was significantly higher than the RC group (HR 0.82[0.71–0.95], *p* = 0.009). However, the heterogeneity was considered substantial with significant result (I^2^ = 67%, *p* < 0.0001). To further analyze the bias of the study, we presented a funnel plot. The funnel plot was not symmetrical, that might be caused by publication bias, as studies with negative or insignificant results were not published. We only used PubMed as database on this study so that there might be some studies were not included. We did not include studies written in languages other than English, thus might favor a language bias. To minimize reporting bias, it is highly recommended to seek key unpublished information in a systematic way. This study also shown a substantial heterogeneity, which might be caused by the difference of tumor stage before NAC. The majority of the studies included patients with organ-confined-disease, except for one study which include N1-3 disease. The regimens of NAC given to the patients varied, which might be the cause of heterogeneity as different regimens of NAC could result in different outcomes. Some of the studies were also considered small, as it only comes from single institution with small samples for each group (less than 100 samples), while there were also studies with thousand samples. A further regression analysis using Egger’s test could be performed to analyse the significance of the bias, rather than a correlation. This result is similar to another meta-analysis which showed significant improvement in the NAC + RC group overall survival (HR 0.87 [0.79–0.96]) [3]. In a meta-analysis conducted by Fahmy et al., the administration of NAC was associated with better 5-year overall survival (60.6% versus 49.1%, *p* = 0.025) [28]. Yin et al. also found that Cisplatin-based NAC gave significant benefit in overall survival (HR 0.87 [0.79–0.96]) [3]. However, Li et al. failed to reject no difference in overall survival between patients received Cisplatin-based NAC followed by RC and RC alone (HR 0.92 [0.84–1.00], *p* = 0.056). The subgroup analysis of three different regimen of Cisplatin-based NAC also showed no significant difference in overall survival between patients who received NAC followed by RC and RC alone [2]. This difference possibly caused by the differences in the studies included in the analysis, as we concluded studies using GC as one of Cisplatin-based regimen for NAC and also studies using non-Cisplatin-based regimen. The overall survival outcome of NAC + RC was mainly affected by bladder cancer stage. In a latest retrospective study conducted by Lane et al. in 2018, patients with organ-confined disease (pT2N0M0) in NAC + RC group had better overall survival in a log rank test (*p* = 0.001) and Cox analysis (*p* = 0.02) compare to patients in RC-only group. However, in pT3-4N0M0 patients, this difference was significant in a log rank test (*p* = 0.01) but not significant in Cox analysis (*p* = 0.30). In addition, another study conducted by Hinata et al. showed that patients with T3-4 stage had worse overall survival than patients with organ-confined disease (*p* < 0.01) [17].

NAC + RC treatment improved the overall survival as it caused a better pathological outcome, with higher rate of pathological downstaging and lower rate of progression into non-organ-confined disease. Nitta et al. analyzed the pathological outcomes of < pT2 stage, with the result that NAC group had a significantly higher rate of < pT2 stage with T2 stage than the non-NAC group [23]. Hermans et al., Martinez-Pineiro et al., and Ploussard et al. stated that proportion of complete pathological downstaging was higher in NAC group compared to upfront RC for both T2 stage and T3-4 [18, 24, 27]. This was associated with better survival rate (*p* = 0.0142) compared to patients without similar pathological downstage results [18]. Vetterlein et al. stated that patients who received NAC were less likely to progress into non-organ-confined disease in subgroups of bladder cancer type compared to RC only group. Most of the studies included in this meta-analysis which presented the median survival for both groups showed that NAC + RC groups had longer median survival compared to RC only group [14, 18], as well as the overall survival [19, 21].

Other study conducted by Yin et al. showed that GC had similar efficacy in improving overall survival outcome compared to MVAC. However, GC was associated in significant lower overall survival than MVAC (HR 1.26[1.01–1.57], *p* = 0.04, I^2^ = 0%) [3]. A study by Peyton et al. also stated that even though GC regimen is the most frequent prescribed NAC, patients using dose-dense MVAC showed better outcomes in any downstaging, including to T0 [29]. However, GC had one advantage compared to MVAC, it had lower toxicity. There were outcome differences based on subjects’ response to NAC. Patients with complete or partial response to NAC had better overall survival outcome compared to patients with no response. To accurately predict the pathological downstaging, a minimal 2 cycles of NAC should be given [8]. The stage evaluation at cycle 2 or less of the NAC was shown not to be effective in the assessment [8].

NAC toxicities has been hypothesized to be the barrier to RC in the MIBC patients. However, these toxicities were not associated with the higher complication rates and considered as self-limiting cases [19]. Some studies showed that NAC regimens were well tolerated by most patients [19]. Retrospective research conducted by Milenkovic et al. about complication rates between NAC + RC and RC only group. The short-term complication rate within 30 days was not significant in both groups (69% and 66%, respectively). Some complications included in the analysis were gastrointestinal,genitourinary, and wound-related complications. Advanced age and comorbidities were the predisposition factors related to morbidity after the surgeries [19].

Despite the significant benefit of NAC + RC in clinical trials and meta-analysis, NAC remains underused in the clinical setting [3]. Several studies revealed that only 1.4%-20.9% patients with MIBC were given NAC before RC [16]. This situation occurred because most urological oncologists consider the patients’ age and comorbidities as obstacles to administer NAC safely in MIBC patients [30]. In elderly patients, administration of NAC was associated with increased toxicity because of medical comorbidities and geriatric syndromes [31]. Several comorbidities that restrain the administration of NAC are renal impairment, poor performance status, and symptomatic disease [9]. The recommended cisplatin-based regimen was contraindicated in 40% of patients with MIBC because of its nephrotoxicity [16]. If the renal function is inadequate, it is recommended to proceed immediately to surgery rather than giving suboptimal dose of cisplatin-based regimen, as suboptimal regimens have no benefit compared to RC alone.

The possibility of definitive treatment delay is another reason most urologists would not give NAC. Despite this presumption, studies have shown that NAC can be administered in a short time using dose-dense regimen with less toxicity and at least the same efficacy as the standard regimen [3]. The adverse effects of chemotherapy such as myelosuppression, gastrointestinal side effects, and nephrotoxicity are also well known. However, these adverse effects did not affect the decision to  perform a RC. The result of SWOG 8710 suggests that RC rates in NAC + RC group were the same as RC alone group (82% versus 81%) [22], with cancellation due to the medical reasons [12]. This reason was lower in NAC + RC compare to RC only group, despite some adverse effects in the NAC + RC group as mentioned previously. Beside urologist preferences or medical reasons, patient preferences also contribute to NAC being underused in clinical settings [32, 33] The low rate of NAC administration was associated with minority races, lower incomes, insurance status, and patients in low-volume hospitals [33].

The delay of NAC has been one of the problems associated with MIBC management. In an investigation performed by Audenet et al., the median time from diagnosis to administration of NAC was 39 days (interquartile range 26–56) [34]. Some risk factors associated with NAC delay were treatment in academic facilities,black race, and patients with Medicaid or other government insurance [34]. This delay caused tumor upstaging, lymph node involvement, and psychological disturbances in the patients [34]. The administration of NAC should be initiated as soon as possible in less than 8 weeks from the time of diagnosis because further delay was associated with increased upstaging risk [34]. The limitation of this meta-analysis was the inclusion of only published literature without detailed personal patient data, including the regimen of NAV given to the patient. We included studies with N1-3 disease, which may result in heterogeneity We also included studies with many variations of NAC regimen and did not conduct a subgroup analysis  by regimen.

## Conclusion

NAC + RC significantly improves overall survival in MIBC patients compared to RC alone. A further study assessing side effects and quality of life regarding NAC is needed to establish a strong recommendation regarding this therapy.

## Data Availability

The studies were available online.

## Abbreviations

MIBC: Muscle invasive bladder cancer
RC: Radical cystectomy
NAC: Neoadjuvant chemotherapy
MVAC: Methotrexate/vinblastine/doxorubicin/cisplatin
GC: Gemcitabine/cisplatin
GCarbo: Gemcitabine/carboplatin
